# Concrete Elastic Modulus Experimental Research Based on Theory of Capillary Tension

**DOI:** 10.3390/ma15103734

**Published:** 2022-05-23

**Authors:** Fengbin Zhou, Changwang Su, Daifeng Wu, Jianmin Hua, Lepeng Huang, Qiming Luo, Maoyi Liu, Mi Nie, Chunyao Tang

**Affiliations:** 1 School of Civil Engineering, Chongqing University, Chongqing 400045, China; 20141613158@cqu.edu.cn (F.Z.); suchangwang@cqu.edu.cn (C.S.); huang_lepeng@cqu.edu.cn (L.H.); luoqiming@cqu.edu.cn (Q.L.); tangchy@cqu.edu.cn (C.T.); 2 Chongqing Urban Investment Infrastructure Construction Co., Ltd., Chongqing 400045, China; 13896090632@139.com (D.W.); liumaoyi@163.com (M.L.); niemi325@139.com (M.N.)

**Keywords:** concrete elasticity modulus, free shrinkage, theory of capillary tension, restraint stress, early cracking

## Abstract

The risk of cracking in the early stage is a critical indicator of the performance of concrete structures. Concrete cracked when the tensile stresses caused by deformation under restraint conditions exceeded its tensile strength. This research aims at an accurate prediction of shrinkage cracking of concrete under constraints. Based on the theory of capillary tension under the concrete shrinkage mechanism, the method to test and compute the elastic modulus of a micro-matrix around the capillary, $E_{t}$, was derived. Shrinkage and porosity determination tests were conducted to obtain the shrinkage values and confining stresses of concrete at different strength grades, different ages and under different restraint conditions, accordingly. Meanwhile, the proposed method of this research was used to obtain $E_{t}$. The restraint stress given by $E_{t}$ was compared with the experimental result under the corresponding time. The results suggested a positive correlation between the elastic modulus of a micro-matrix around the capillary, $E_{t}$, precomputed by the theory, and the static elastic modulus, $E_{c}$, and that the ratio between the two gradually decreased with the passage of time, which ranged from 2.8 to 3.1.

## 1. Introduction

The cracking of concrete has long been an issue puzzling many scientific researchers and engineering personnel. With the deepening of relevant research and the improvement of concrete mechanical properties, the cracking of concrete has seldom arisen from the impact of the direct load under normal use. Nevertheless, indirect impact, including the impact of temperature on the large-volume concrete as well as the creep and auto-shrinkage of concrete, has gradually played an important role in causing the cracking of concrete [1]. Research suggests that more than 80% of the concrete structural cracks have been resulted from the indirect impact, among which the impact of shrinkage is the most significant [2,3].

Prior research into the shrinkage performance of concrete mainly focused on the impact of materials [4,5,6], temperature and humidity [7], and internally confined reinforcing steel bars (diameter [8], distribution [9,10], materials [11], prevention methods [12,13] and surface form [14]) on the concrete-free shrinkage. As to concrete cracking triggered by indirect load, scholars are interested in examining this issue from the perspective of plain concrete [15] and internally confined concrete [16]. The research suggests that, no matter which constraint status the concrete is under, as long as the restraint stress imposed on the concrete is stronger than the tensile strength of the concrete itself, then the concrete will crack [11]. Thus, accurate computing of the concrete restraint stress is a critical means to predict the early cracking of concrete [17]. As one of the important mechanical properties, the elastic modulus can well represent the deformation characteristics of concrete, and is an important condition for calculating the restraint stress of concrete.

Many scholars have carried out a series of studies on the elastic modulus of concrete. Several such studies have found that the elastic modulus has a great influence on the properties of concrete materials, such as railway sleepers [18] and precast concrete slabs [19]. There were also some other studies focusing on the influencing factors of the elastic modulus of concrete at the macroscopic level [20,21]. For example, Wang [22] studied the effect of aggregate content of different particle sizes on the elastic modulus in experiments, and proposed a model that can effectively predict the elastic modulus of recycled aggregate concrete. Nath [23] studied the change of the elastic modulus of polymer concrete under different curing conditions. There were also more and more studies focusing on the microscopic properties of concrete, trying to discover the mechanism of its influence on the macroscopic properties. In recent years, with the development of microscopic testing techniques such as nanoindentation, the relationship between the microscopic phase and the macroscopic elastic modulus of concrete has received special attention [24,25,26]. In addition, the accurate acquisition and prediction of the elastic modulus of concrete was also a research focus, especially for modern high-performance concrete structures with early loading for rapid construction. The prediction method of the concrete elastic modulus was mainly the effective modulus [27], age-adjusted effective modulus [28] and the Trost–Bazant method [29]. The effective modulus method was reported to be only suitable for non-ageing concrete subjected to constant stress history; therefore, it has rather limited applicability in practice [27]. The age-adjusted and Trost–Bazant model parameters were calibrated by tests on mature concrete, thus the suitability of the above models for predicting tensile creep was questioned early on when concrete properties changed rapidly with time [30,31]. In terms of obtaining the elastic modulus of concrete, $E_{C}$ was measured by the method recommended by the “Standard for test methods of concrete physical and mechanical properties” [32]. The way to measure $E_{C}$ is specified below in great detail:

The standard prism (standard dimensions: 150 × 150 × 300 mm) test specimens are employed to measure the concrete axial compressive strength ($f_{cp}$). The upper limit for loading stress is 1/3$f_{cp}$. After the loading and unloading are repeated for five times (each time lasting for around one to two minutes), the stress–strain curve thus measured is basically linear. The slope rate of the curve is used to denote the concrete elastic modulus, $E_{C}$. $E_{C}$ can be specified as below:

(1) $$E_{c}=\frac{F_{a}-F_{0}}{A}\times \frac{L}{\Delta n}$$

(2) $$\Delta n=\varepsilon_{a}-\varepsilon_{0}\;$$

where $F_{a}$ denotes the load (N) when stress reaches one third of the axial compressive strength, $f_{cp}$; $F_{0}$ indicates the initial load (N) when stress is 0.5 MPa; A represents the bearing area (mm^2^) of the specimen; L denotes the measurement gauge length; ∆*n* indicates the average (mm) of the deformation on two sides of the specimen when the load $F_{0}$ is increased to $F_{a}$; $\varepsilon_{a}$ is the average (mm) of the deformation on two sides of the specimen when the load is $F_{a}$; $\varepsilon_{0}$ denotes the average (mm) of the deformation on two sides of the specimen when the load is $F_{0}$. 

Among the aforesaid methods, the loading process is rapid, which can be completed generally in around 5 to 10 min. However, the literature suggests that the concrete elastic modulus and the speed imposed on the load are closely linked [33]. The faster the loading speed is, the larger the concrete elastic modulus is. When the issue of concrete shrinkage cracking is studied, the concrete shrinkage stress (which is responsible for concrete shrinkage) is imposed over a slow, constantly changing and long-term process (the effect of the shrinkage stress can last for around 100 days or even longer) [34]. Since the loading rate of the concrete static compressive elastic modulus obtained by the aforesaid experimental approach is relatively fast, the concrete constrained stress given by the concrete static compressive elastic modulus thus obtained might not be static, which might expose the concrete to a high risk of cracking under the conventional reinforcement ratio. This is inconsistent with the phenomenon observed in the engineering process [35].

Based on the theory of capillary tension, this research proposes a test and calculation method for the elastic modulus of a micro-matrix around the capillary, which can solve the problem of inaccurate elastic modulus measurement caused by the acceleration of the loading, and can be used to test the elastic modulus of the concrete on a specific time and at a specific place under special conditions and to accurately calculate the constrained tensile stress of the concrete and predict the cracking of the concrete. Experiments were carried out to test the concrete shrinkage stress under different constraints as well as at different strength grades and different ages. Through theoretical deduction and experiments, the elastic modulus of a micro-matrix around the capillary, $E_{t}$, can be worked out based on the theory of capillary tension. With $E_{t}$, the concrete constrained stress in constrained specimens can be further calculated. The constrained stress thus obtained was compared with the concrete tensile strength of the corresponding moment so as to obtain the predicted moment for the cracking of the concrete specimen under different constraints. The cracking moment thus obtained compared with the real cracking moment in the experiment, which suggested that $E_{t}$ can correctly predict the moment for cracking the concrete.

## 2. Theoretical Model

According to previous research findings, the capillary strain is a main reason for concrete shrinkage [36]. Therefore, this research proposed a method to calculate the elastic modulus of a micro-matrix around the capillary that is obtained by the correlation between the concrete capillary tension and free shrinkage.

Assuming that the concrete longitudinal length is far larger than the length of other directions, the capillary tension and auto-shrinkage of other directions can be omitted. Under this condition, the stress–strain correlation on the longitudinal length should be taken into consideration, namely the stress correlation between the capillary tension and the free auto-shrinkage strain. The capillary tension and the free shrinkage strain at the moment of $t_{1}$ and $t_{2}$ can be obtained by testing and computing. This meant that the average of the elastic modulus of a micro-matrix around the capillary, $E_{t}$, between $t_{1}$ and $t_{2}$ could be obtained, which could indicate $E_{t}\left(\Delta t\right)$ within the time of Δt. Below is the specific approach:

First, the mercury injection apparatus was used to measure the concrete pore structure at the moment of $t_{1}$ so as to obtain the capillary tension, $\sigma_{c}\left(t\right)$, as the driving force that triggered the concrete-free shrinkage. The computing formula can be written, as below [37]:

(3) $$\sigma_{c}\left(t\right)=\Delta P=\frac{2\gamma \cos\theta }{r\left(t\right)}$$

where γ denotes the surface tension of the capillary internal wall, which is 7.28 × 10^−2^ N/m at the temperature of 20 °C; *θ* represents the contact angle of the liquid and solid interface (which is zero in the case of concrete); r stands for the critical capillary diameter of the concrete at the moment of t.

At the same time, the concrete shrinkage stress and the concrete-free shrinkage stress at the moment of $t_{1}$, namely $\sigma_{c}\left(t_{1}\right)$ and $\varepsilon_{f}\left(t_{1}\right)$, respectively, can be obtained through the free shrinkage experiment. After the moment of ∆*t*, the concrete shrinkage stress and the concrete-free shrinkage stress at the moment of $t_{2}$, namely $\sigma_{c}\left(t_{2}\right)$ and $\varepsilon_{f}\left(t_{2}\right)$, respectively, can be obtained by Equation (3) and the free shrinkage experiment.

**Figure 1:**
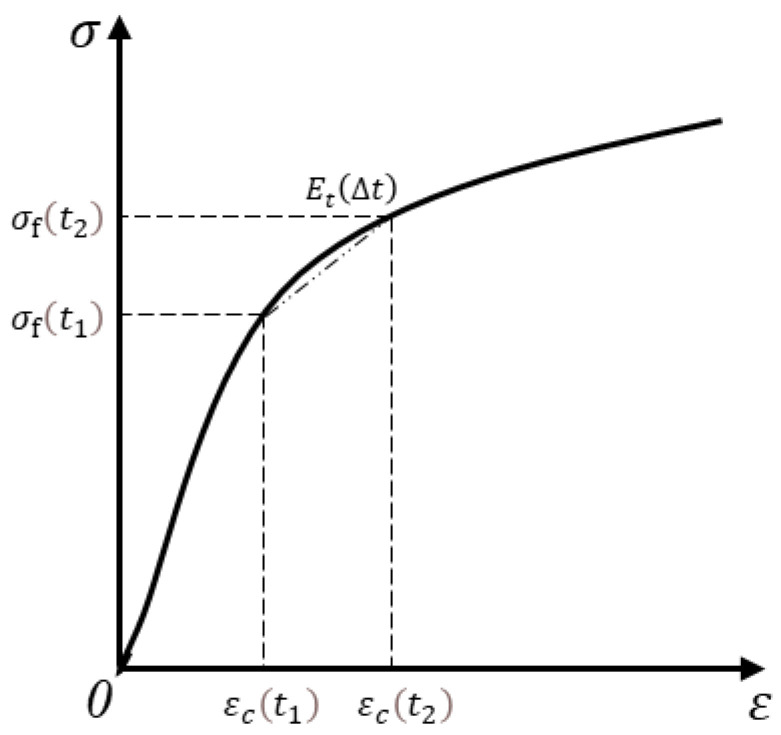
Schematic diagram of the elastic modulus of a micro-matrix around the capillary ($E_{t}$).

Thereby, as shown in Figure 1, the elastic modulus of a micro-matrix around the capillary, $E_{t}\left(\Delta t\right)$, over the Δt period of time at the moment of $t_{2}$, can be obtained:

(4) $$E_{t}\left(\Delta t\right)=\left(\sigma_{c}\left(t_{2}\right)-\sigma_{c}\left(t_{1}\right)\right)/\left(\varepsilon_{f}\left(t_{2}\right)-\varepsilon_{f}\left(t_{1}\right)\right)$$

Of special note is that $\sigma_{c}\left(t_{2}\right)$, $\sigma_{c}\left(t_{1}\right)$, $\varepsilon_{f}\left(t_{2}\right)$ and $\varepsilon_{f}\left(t_{1}\right),$ measured by the experiment, are arranged at the interval of Δt, namely Δt = $t_{2}-t_{1}$. Therefore, the concrete relativeelastic modulus under the capillary tension can be written as the average elastic modulus over the Δt period of time.

**Figure 2:**
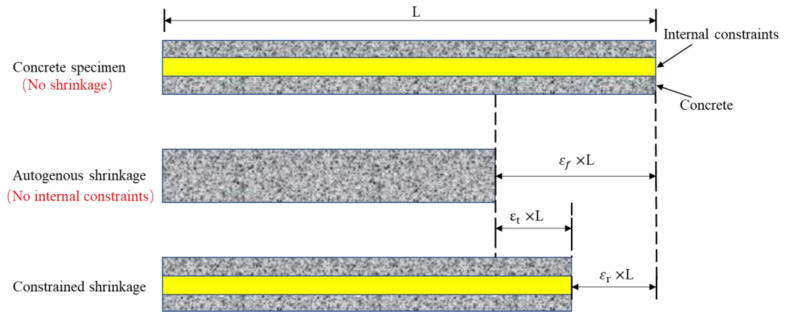
Schematic diagram of constrained shrinkage in concrete.

As shown in Figure 2, when the concrete is in free shrinkage, the strain of the concrete is $\varepsilon_{f}$ compared to the original concrete specimen. When the concrete is subject to the restrain caused by the internal steel bars or steel plates, the concrete will shrink compared to the steel bars or steel plates, and the steel bars will exert a force that could inhibit the shrinkage of the concrete, so that the shrinkage of the concrete is limited to a certain extent. The ultimate extent of shrinkage of the concrete is $\varepsilon_{r}$. Therefore, in the concrete specimen, the restrained tensile strain can be expressed as:

(5) $$\varepsilon_{t}=\varepsilon_{f}-\varepsilon_{r}$$

where $\varepsilon_{r}$ is the shrinkage stress of the constrained concrete specimens; $\varepsilon_{f}$ denotes the concrete shrinkage stress, which can be obtained through the free shrinkage experiment. 

Then, the restraint stress within the concrete at certain moment, t, can be written as below:

(6) $$\sigma_{c}\left(t\right)=\varepsilon_{t}\left(t\right)\times E_{t}\left(\Delta t\right)$$

When $\sigma_{c}\left(t\right)\;$ is larger than the concrete tensile strength of the *t* moment, the concrete cracking will occur.

## 3. Experimental Research

**Figure 3:**
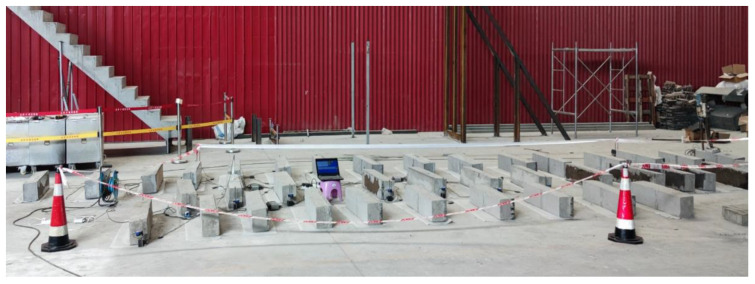
Schematic diagram of the field test situation.

In order to obtain the concrete elastic modulus of a micro-matrix around the capillary, $E_{t}$, and verify the accuracy of $E_{t}$ in predicting the concrete internal stress status and cracking, corresponding experiments were carried out. The test considered three influencing factors of different concrete strength grades, different concrete ages and different restraint conditions. From the perspective of the static elastic modulus of concrete, different grades of concrete had large differences in the static elastic modulus, thus, in this test, the concrete strength grade was used as the influencing factor. Given the time-varying properties of concrete, the age of concrete was also one of the main influencing factors. From the perspective of concrete shrinkage, while helping the concrete to bear external loads, the steel bars also constrained the free shrinkage of the concrete because it did not deform itself, thus generating constraining stress inside the concrete. Judging from a large number of engineering cracking phenomena, the restraint stress generated by steel bars on concrete was an important reason for the cracking of reinforced concrete structures during the construction. In this test, in order to make the concrete crack, the steel bars were replaced by steel plates, which were of higher restraint so as to facilitate the observation of the test phenomenon. Figure 3 shows a schematic diagram of the field test situation.

### 3.1. Materials for Experiments

Portland cement (P.O42.5R) and Bolei Level-I fly ash and additive were adopted as experimental materials. Their chemical components and mechanical properties are demonstrated in Table 1 below. Sand and gravel of the pebble stone mechanism were adopted as aggregates. At the same time, the polycarboxylate superplasticizer ZJC-01 was used. The steel plate adopted was Q235 steel.

### 3.2. Experimental Design

The mixing ratio of concrete (C30, C40, C50 and C60) at four different strength grades is presented in Table 2 below.

Concrete shrinkage stress, pore structure and concrete relative elastic modulus under the capillary tension at different strength grades, ages and constraints can be measured and calculated. In this research, two types of specimens were designed. The first type was the concrete cracking specimen constrained by steel plates (the steel plate thickness was 20, 40 and 60 mm, respectively, and the steel plate lengths were all 1000 mm, all of which were placed at the bottom within the modulus). The second type was the concrete auto-shrinkage specimen without the constraint of steel plates. The dimensions of these two types of concrete specimens were both 200 mm × 200 mm × 1000 mm. To facilitate the observation of concrete cracking, dry preservation was used. The mold was made up of glass, which was convenient to demolish. The screws and angle steel were used for fixture around the organic glass mold. The upper of the mold is clipped with timbering support to prevent swelling. Table 3 provides specifications of different test specimens.

### 3.3. Shrinkage Test

**Figure 4:**
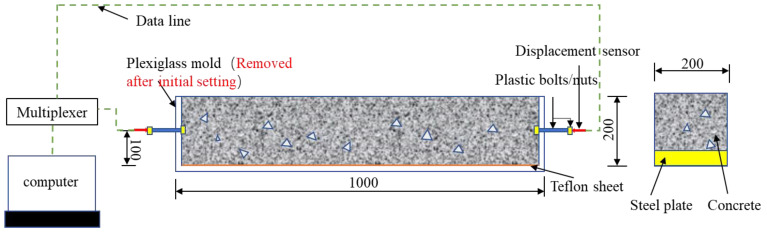
Concrete shrinkage, internal temperature and humidity measuring devices (dimensions in mm).

Concrete shrinkage test usually adopts the commonly used auto-shrinkage test system. Figure 4 prevents an overview of the concrete shrinkage displacement, temperature and humidity test system. After the initial setting of concrete, the organic glass mold was demolished, and the 1 mm-thick Teflon plate was used to cover the test specimen bottom to ensure the test specimen could move freely in the surroundings.

Auto-shrinkage test specimens were measured with the linear variable differential transformer (LVDT) installed in the center of the two ends of the test specimens. The measurement precision and scope were set to be 1 μm and 2 mm, respectively. In order to ensure the accuracy of LVDT, two nuts were placed in the center of the two ends of every test specimen. After the initial setting of concrete, the organic glass mold was removed, and the two plastic bolts were both screwed into the two nuts. After that, the LVDT test bar had a direct contact with bolts. The computer was used to automatically measure the records, and the experiment measured the shrinkage of all plain concrete as well as the shrinkage of test specimens inbuilt with the steel plate, whose thickness is 20 mm, 40 mm and 60 mm, respectively. The impact of temperature on concrete could be measured through a preliminary test. Specifically, the temperature and humidity sensor were built into every sample, and all test specimens were placed under the environment at the temperature of 20 ± 1 °C and the relative humidity of 60 ± 5%. There was one specimen per group for the shrinkage test.

### 3.4. Experiment for Measurement of Concrete Pore Rate

Similar to the concrete shrinkage strain measurement experiment, concrete test specimens poured in-place by four mixing ratios (C30, C40, C50 and C60) were adopted to test the concrete porosity. When the concrete age reached 2, 3, 4, 5, 7, 14 and 28 days, respectively, the core samples were extracted from test specimens to test the concrete porosity. The porosity test had three specimens per group.

**Figure 5:**
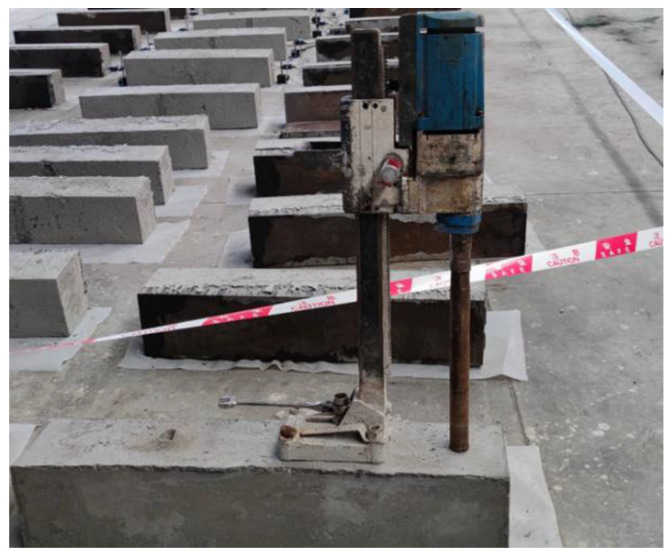
Core sampling method.

The concrete core samples thus extracted were first crushed and then screened to have concrete particles collected. The diameter of these concrete particles ranged from 2.5 mm to 5 mm. The acetone was used to prevent the hydration of concrete, and the vacuum dryer was applied to the dry samples. At last, the AutoPoreIV9510 device was employed to test the concrete porosity. Figure 5 shows the schematic diagram of the core sampling test.

### 3.5. Cracking Observation

The cracking observation experiment was conducted to measure the early cracking of all concrete constrained by steel plates (steel plates of three different kinds of thicknesses were placed in four kinds of concrete prepared by four mixing ratios, respectively. To ensure the accuracy of the observation, each kind of concrete had three test specimens which were the same, thus there were 48 test specimens in total).

The reading microscope, which can amplify the target by 40 times (division value was 0.01 mm), was adopted to observe the concrete surface. The time of the first tensile crack developed on all test specimens constrained by steel plates, together with the follow-up changing the breadth and length of the crack development, was put down.

### 3.6. Basic Performance of the Experimental Group

The mechanical properties of all steel plates adopted for this research are presented in Table 4. The mechanical performance experiment of test specimens mainly included the cubic compressive strength when the concrete curing age was 3, 7, 14 and 28 days, respectively, with the static compressive elastic modulus and the splitting tensile strength on the 2nd, 3rd, 4th, 5th, 7th, 14th and 28th day, and 3 specimens were tested in each group. The basic parameters are shown in Table 5.

## 4. Results and Discussions

### 4.1. Concrete Shrinkage Deformation

**Figure 6:**
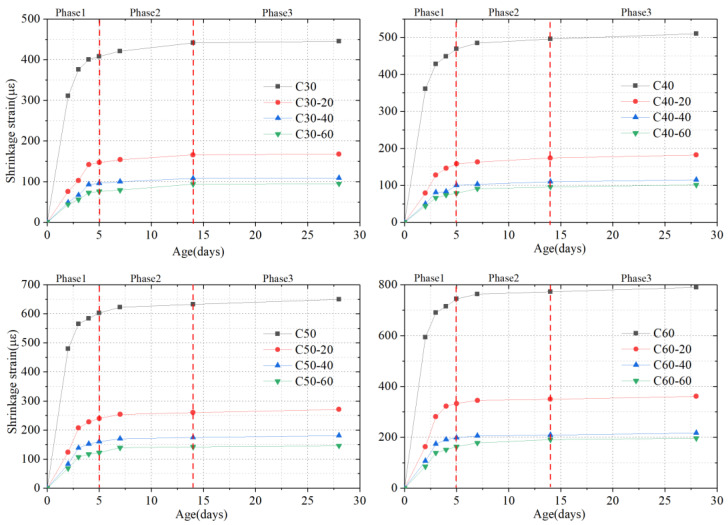
Concrete shrinkage curve changing with time.

Figure 6 displays the shrinkage curve of different test specimens over 28 days. The curve-changing rules demonstrate that the free shrinkage test specimens and the test specimens constrained by steel plates all undergo the following three stages. At the first stage (1–5 days), driven by the strong hydration reaction of concrete, the shrinkage increased rapidly. At the second stage (5–14 days), with the weakening of the concrete hydration, the shrinkage slowed down. At the third stage (14–28 days), the hydration effect was further weakened, leading to a decline of the curve slope. Then, the shrinkage slowed down again, which finally became basically stable. On the whole, for either the plain concrete experiment or the constrained concrete test specimen, the concrete shrinkage variations were increasingly significant with the increase of the age. For example, the shrinkage of C30 plain concrete test specimens increased from 311 με on the 2nd day to 445 με on the 28th day. As to the C30 shrinkage test specimen constrained by 20 mm steel plates, its shrinkage rose from 76 με on the 2nd day to 168 με on the 28th day. Either for the plain concrete test specimens or the test specimens constrained by plain plates, the shrinkage value could always be ranked by the following order, namely C60 > C50 > C40 > C30, which was basically consistent with the phenomenon observed by prior researchers [2,37,38].

As shown in Figure 6, steel plates after being placed in concrete test specimens of different strengths were all obviously constrained, resulting in the decrease of shrinkage at every stage. For example, the C30 plain concrete test specimen shrinkage was 445 με on the 28th day. After 20 mm-tick steel plates were added, the shrinkage of the constrained test specimens was 168 με, which was reduced by 277 με. In terms of C60 test specimens, the shrinkage of the plain concrete test specimens was 790 με on the 28th day. After 60 mm-thick steel plates were added, the shrinkage was 196 με on the 28th day, which dropped by 594 με. Additionally, steel plates of different thicknesses can exert different degrees of the constraining effect on concrete. The thicker the steel plate is, the stronger the constraining effect of the steel plate is on the concrete, and the lower the shrinkage of concrete. Take C40 concrete, for example. When the shrinkage of any moment was chosen for comparison, the same results could be obtained, including C40 > C40-20 > C40-40 > C40-60. Other concrete strengths were found with the same phenomenon. Moreover, the curves of different steel plates were compared, demonstrating that with the increase of the steel plate thickness, the impact of the steel plate thickness on the concrete shrinkage would gradually weaken. Take C50 concrete, for example. The shrinkage of C50 concrete without adding steel plates was 650 με on the 28th day. After 20 mm-thick steel plates were added, the shrinkage of C50 concrete was 271 με on the 28th day. After 40 mm-thick steel plates were added, the figure changed to 181 με. After 60 mm-thick steel plates were added, the figure changed to 146 με. The shrinkage difference between C50 and C50-20 on the 28th day was 379 με; the shrinkage difference on the 28th day between C50-20 and C50-40 was 90 με; the shrinkage difference on the 28th day between C50-40 and C50-60 was 35 με. It can be found that, as the steel plate thickness increased, the shrinkage difference decreased, accordingly.

### 4.2. Concrete Pore Structural Parameters

Table 6 presents the pore structural parameters of samples on the 2nd, 3rd, 4th, 5th, 7th and 14th day, respectively. The pore structural parameters included the critical capillary diameter, average pore diameter, median pore diameter and pore diameter distribution. As the time of concrete curing increased, the critical capillary diameter, average pore diameter and median pore diameter of concrete of different strengths all decreased considerably. Take C30 concrete, for example. Its critical capillary diameter on the 3rd, 7th, 14th and 28th was 91.00 nm, 35.86 nm, 24.23 nm and 22.71 nm. After observing the pore structural size, one could observe that the small pore diameter in concrete takes up an increasing percentage, while the percentage of the large pore diameter decreases. Take C50 concrete, for example. The pore structure that is larger than 100 nm took up 34.31%, 11.55%, 8.09% and 5.51% on the 3rd, 7th, 14th and 28th day, respectively. The concrete pore diameter decreased for two main reasons: (1) As the concrete curing time lengthened, the cement hydration effect continued. In this process, the product of the cement hydration effect could make up the microscopic cracks and pores in the concrete structure. (2) During the cement hydration process, the water in the concrete pore was constantly consumed. Because of the consumption of water in the pore, the capillary tension was imposed on the concrete pore wall. Affected by the capillary tension, the distance between concrete pores was constantly reduced, which resulted in the decrease of various pore structural parameters.

Additionally, the critical capillary diameter of concrete of different grades was compared, revealing that, under the same concrete age, the higher the concrete grade was, the smaller the critical capillary diameter of concrete would be. Take C30, C40, C50 and C60 concrete, whose age was 28 days, for example. Their critical pore diameter was 22.71 nm, 18.64 nm, 17.29 nm and 15.15 nm, respectively. This was because the concrete content was higher in concrete of a higher grade, and the cement hydration effect within the concrete was more complete, thus ensuring a more compact internal structure for the concrete.

### 4.3. Materials for Experiments

Table 7 presents the capillary tension on concrete test specimens of different grades and at different ages. As one observes in Table 7, as the age increased, the capillary tension of concrete test specimens of different grades all increased correspondingly. For example, the capillary tension on C30 increased from 1.42 MPa on the 3rd day to 6.41 MPa on the 28th day. The capillary tension on C40 increased from 1.51 MPa on the 3rd day to 7.81 MPa on the 28th day. As the concrete curing time increased, the cement hydration effect could, to some extent, make up the microscopic cracks and pores in the concrete structure. The capillary pore diameter in concrete decreased. The capillary tension thus obtained was larger. Additionally, when the age was the same, the higher the concrete grade was, the higher the capillary tension was imposed on the concrete. Take C30, C40, C50 and C60 concrete at the age of 28 days, for example. The capillary tension imposed on these four kinds of concrete was 6.41 MPa, 7.81 MPa, 8.42 MPa and 9.61 MPa, respectively. The higher the cement content was in concrete of higher grades, the more complete the cement hydration effect was inside the concrete, and the more compact was the concrete internal structure. This meant that the smaller the capillary pore diameter was, the higher the capillary tension.

### 4.4. Calculation of the Elastic Modulus of a Micro-Matrix around the Capillary

**Figure 7:**
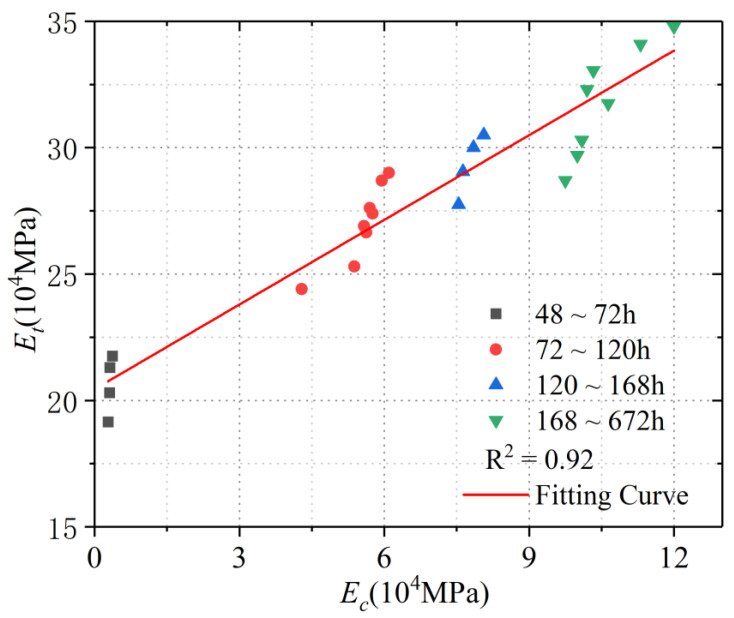
Fitting curve of elastic modulus of a micro-matrix around the capillary ($E_{t}$) and static elastic modulus ($E_{c}$).

In this experiment, the porosity and static (static compression) elastic modulus of concrete under different statuses were measured on the 2nd, 3rd, 4th, 5th, 7th, 14th and 28th, respectively. The capillary tension and free shrinkage strain measured at different moments were used to work out the elastic modulus of a micro-matrix around the capillary, $E_{t}$. Figure 7 was drawn to present the development trend of the elastic modulus of a micro-matrix around the capillary. The elastic modulus of a micro-matrix around the capillary, $E_{t}$, can reflect the slope of the secant line in Figure 1, which can indicate the elastic modulus of a micro-matrix around the capillary over the period of time.

**Figure 8:**
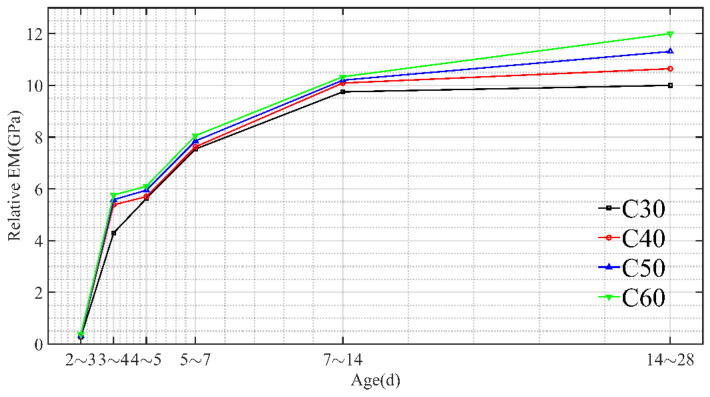
The development trend of the elastic modulus of a micro-matrix around the capillary ($E_{t}$).

As shown in Figure 8, as the concrete age increased, the elastic modulus of a micro-matrix around the capillary kept on increasing. The elastic modulus of a micro-matrix around the capillary growth of concrete was within 168 h. After 168 h, the elastic modulus of a micro-matrix around the capillary was gradually stabilized. On the whole, consistent with the changing rule of the concrete elastic modulus under static compression, the elastic modulus of a micro-matrix around the capillary increased rapidly in the first 7 days but slowed down after 7 days and finally became stabilized. Similarly, the higher the concrete strength was, the higher the elastic modulus of a micro-matrix around the capillary of concrete.

The elastic modulus of a micro-matrix around the capillary, $E_{t}$, of concrete was compared with the static elastic modulus, $E_{c}$, measured by this research. Since the elastic modulus of a micro-matrix around the capillary needed two static elastic modulus to be worked out, the average of the two static elastic moduli was compared with the elastic modulus of a micro-matrix around the capillary. For example, the elastic modulus of a micro-matrix around the capillary worked out from 48 h to 72 h was compared with the average static elastic modulus measured from 48 h to 72 h. The comparison results were presented in Table 8. As one notices in Table 8, the ratio of the elastic modulus of a micro-matrix around the capillary to the static elastic modulus was declining. With the passage of time, the ratio of $E_{c}$ to $E_{t}$ changed from 2.8 to 3.1, which basically maintained at around 3.

According to Table 8 above, Figure 7 further displays the fitting curve of the elastic modulus of a micro-matrix around the capillary, $E_{t}$, and static elastic modulus, $E_{c}$. It can be observed that there was a positive correlation between them, and that their correlation coefficient was 0.92. The computing formula after fitting could be obtained and written as below:

(7) $$y=20.44+1.12x\;(x\;\mathrm{denotes}\;E_{c},\;y\;\mathrm{denotes}\;E_{t})$$

### 4.5. Crack Development

Observing the initial cracking time of concrete held vital significance to learn about the concrete cracking development status. In order to study the influence of different constraints on concrete cracking, three kinds of steel plates that had a higher thickness were adopted.

**Figure 9:**
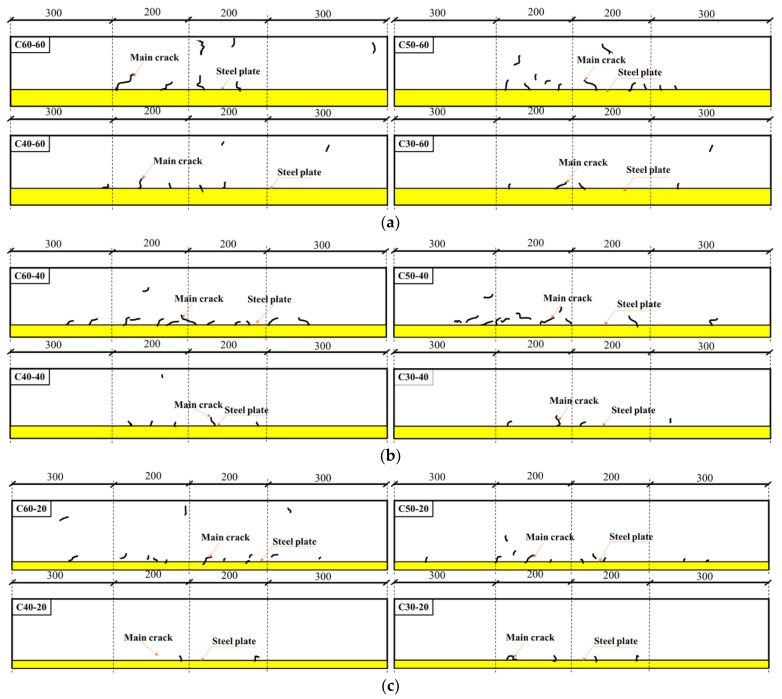
Cracking schematic diagram: (**a**) Steel plate thickness is 60 mm; (**b**) steel plate thickness is 40 mm; (**c**) steel plate thickness is 20 m.

In a bid to better display the crack form and position, the concrete actual crack chart was simplified into Figure 9. The chart shows the cracking models of four kinds of concrete test specimens (C30, C40, C50 and C60) after steel plates of different thicknesses were added. In Figure 9, the yellow area is where steel plates are placed, while the black curve is the concrete crack. As one observes in Figure 9, the main cracks of four kinds of test specimens were concentrated in the middle area and the overall distribution was relatively even. When the steel plate thickness remained unchanged, the higher the concrete strength was, the more the cracks could be found on test specimens, and also the more compactly the cracks would be distributed thereon. When the concrete strength remained the same, the higher the steel plate thickness was, and the more the cracks would be found at the interface between the steel plate and the concrete.

In terms of crack development, C30 test specimens were found with a steady crack development. After cracks appeared in C30 test specimens, no further cracking was observed, and the maximum crack width was very small, which was 0.08 mm on average. The maximum crack length was 32 mm on average. All this was hard to observe by naked eyes. C40 test specimens, after developing the first crack, was found with other tiny cracks, subsequently. Their maximum crack width was 0.165 mm on average, while their maximum length was 37 mm on average. But the crack development was relatively slow. Multiple cracks were found with C60 test specimens, of which major cracks developed fast, with the maximum crack width reaching 0.28 mm and the maximum crack length reaching 63 mm.

Table 9 presents the cracking observation list of concrete of different strengths and constrained by steel plates of different thicknesses. According to data displayed in Table 9, when the concrete strength was given, the thicker the steel plate was, the stronger the constraining effect was, and the larger the maximum crack width and the maximum crack length. Prior research findings suggested [39] that, due to the filling of hydration products, the concrete pore structure dropped. When the reinforcing steel bar constrained the concrete free shrinkage, the constraining of the reinforcing steel bar could lead to the shearing strength at the interface between the concrete and the reinforcing steel bar. Additionally, the normal stress (or named as “restraint stress”), which was opposite to the shrinkage direction, was generated in concrete, and the normal stress was also opposite to the concrete shrinkage triggered by the capillary tension. The restraint stress of the reinforcing steel bar on the concrete increased along with the increasing reinforcement ratio. As the reinforcement ratio increased, the concrete was more vulnerable to cracking.

### 4.6. Concrete Cracking Prediction

The elastic modulus of a micro-matrix around the capillary, $E_{t}$, of concrete over a period of time could be given by Equation (4). At the moment, the restraint stress, $\sigma_{t}\left(t\right)$, should be a range value, which should consider the restraint stress at the start of the period of time and the strain at the end of the time to be worked out. Take the computing process of the C30-20 restraint stress from the 2nd day to the 3rd day, for example. Four parameters, including $\sigma_{c}\left(t_{day3}\right)$, $\sigma_{c}\left(t_{day2}\right)$, $\varepsilon_{f}\left(t_{day3}\right)$ and $\varepsilon_{f}\left(t_{day2}\right)$, were necessary to compute the elastic modulus of a micro-matrix around the capillary, $E_{t}$. When the restraint stress from the 2nd day to the 3rd day was computed, the $E_{t}$ obtained above should be multiplied with the restraint shrinkage $\left(\varepsilon_{f}\left(t_{day2}\right)-\varepsilon_{t20}\left(t_{day2}\right)\right)$ of the 2nd day and the restraint shrinkage $\left(\varepsilon_{f}\left(t_{day3}\right)-\varepsilon_{t20}\left(t_{day3}\right)\right)$ of the 3rd day. Thereby, a range value was obtained.

**Figure 10:**
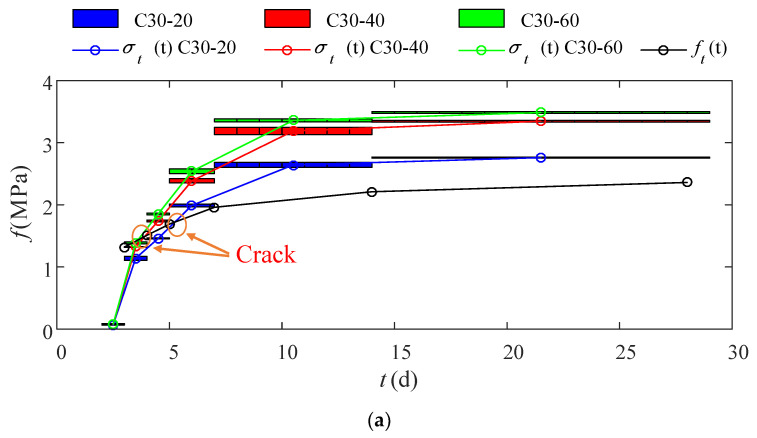

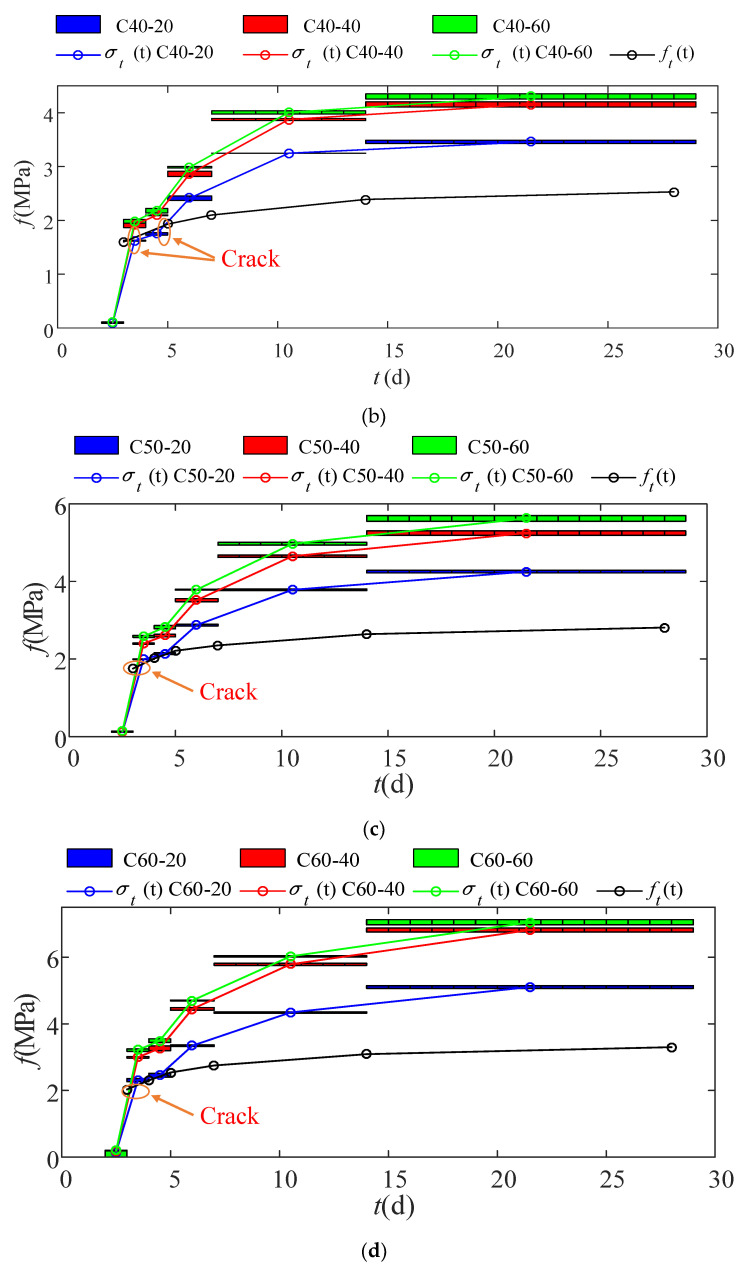
Concrete cracking time schematic diagram: (**a**) C30; (**b**) C40; (**c**) C50; (**d**) C60.

According to $E_{t}$ obtained in the previous part and combining the restraint shrinkage, $\varepsilon_{t}$ was obtained by computing, and the restraint stress, $\sigma_{t}\left(t\right),$ at different initial cracking times, could be obtained by computing. At the same time, the concrete tensile strength, $f_{t}$, of the corresponding time could be obtained by the test. The specific results are shown in Figure 10. The intersecting point between the restraint stress curve and the concrete tensile strength curve marked the cracking time of the concrete. Take the C30 concrete, for example. The C30-40 and C30-60 restraint curve and the concrete tensile strength curve were intersected between the 3rd day and the 4th day. This meant that, under the same constraints, the higher the concrete strength was, the earlier the cracking. Table 10 shows how to compute the restraint stress using the elastic modulus of a micro-matrix around the capillary, $E_{t}$. Thereby, in Figure 10 where the predicted concrete cracking time and the true concrete cracking time were compared, only C30-20 was found with the difference between its predicted cracking time and the true cracking time. All the rest demonstrated good agreement with the predicted results.

## 5. Conclusions

Based on the theory of capillary tension, the method to work out the elastic modulus of a micro-matrix around the capillary was obtained. Meanwhile, concrete constrained cracking experiments were carried out to verify the accuracy of this method. Through the measurement of the pore structure and porosity as well as the observation of concrete cracks, the impact of different steel plate thicknesses and different mixing ratios on the concrete shrinkage, pore structure, capillary tension and cracking was examined. Below are some deductions of the elastic modulus of a micro-matrix around the capillary of concrete and at different mixing ratios.

1. The free shrinkage of test specimens at different mixing ratios was significantly different. The higher the concrete strength was, the higher the shrinkage. Steel plates had an obviously restraining effect on concrete. The thicker the steel plate was, the stronger the restraining effect.
2. Under the prerequisite that other conditions remained unchanged, the smaller the concrete water-cement ratio was, the faster the crack development was after the concrete cracking, and the longer crack width was thus formed.
3. The positive correlation between the elastic modulus of a micro-matrix around the capillary, $E_{t}$, of the concrete and the static elastic modulus, $E_{c}$, of concrete was observed. The ratio between the two gradually decreased with the passage of time, which was finally stabilized within the range from 2.8 to 3.1.
4. The concrete cracking time predicted by $E_{t}$ was close to the real cracking time. This suggested that the proposed method could effectively compute the elastic modulus of a micro-matrix around the capillary and predict the restraint stress, thus finally achieving the goal of the cracking prediction.

Due to the limitation of test conditions and funds for the test, the calculation of the elastic modulus in the article uses data with an interval of 24 h or more. If the sampling interval of porosity and shrinkage values is shortened as much as possible, more accurate relative elastic modulus data can be obtained, and this method can be used to better predict concrete cracking.

## Figures and Tables

**Table 1:** Chemical components and physical characteristics of cementing materials.

Composition% (Mass)	Portland Cement	Fly Ash
SiO_2_	24.36	46.53
CaO	67.35	4.45
Al_2_O_3_	5.48	22.07
Fe_2_O_3_	4.36	15.01
MgO	1.42	1.22
SO_3_	0.54	1.36
Specific surface (cm^2^/g)	3360	4723
Density (g/cm^3^)	3.13	2.35
SiO_2_	24.36	46.53

**Table 2:** Concrete mixing ratios (kg/m^3^).

Mix	Water–Binder Ratio	Water	Cement	Fly Ash	Sand	CA	Polycarboxylate Superplasticizer (PS)
C30	0.56	195	346	70	804	1091	9.0
C40	0.48	170	353	67	762	1083	90.2
C50	0.40	160	401	58	721	1071	11.3
C60	0.32	154	482	48	680	1064	12.7

**Table 3:** List of test specimens.

No.	Strength Level	Thickness of Steel Plates (mm)	Number of Specimens
C30	C30	0	3
C30-20		20	
C30-40		40	
C30-60		60	
C40	C40	0	
C40-20		20	
C40-40		40	
C40-60		60	
C50	C50	0	
C50-20		20	
C50-40		40	
C50-60		60	
C60	C60	0	
C60-20		20	
C60-40		40	
C60-60		60	

Notes: C30-40 refers to the 40 mm-thick steel plate specimens placed in C30 concrete. Other signs can be interpreted by analogy.

**Table 4:** Characteristics of steel plates.

	Material	EM (10^4^ MPa)	Yield Strength (MPa)	Ultimate Strength (MPa)
Steel plate	Q235B	20.6	260.0	460

**Table 5:** Characteristics of steel plates.

Mix Ratio	Age (Days)	Physical Properties		
		Cubic Compressive Strength (MPa)	Splitting Strength (MPa)	EM (10^4^ MPa)
C30	2	-	-	15.6
	3	21.2	1.32	22.7
	4	-	1.51	26.1
	5	-	1.69	27.2
	7	23.1	1.96	28.3
	14	26.9	2.21	29.1
	28	29.6	2.36	30.3
C40	2	-	-	16.7
	3	25.8	1.62	23.4
	4	-	1.81	27.6
	5	-	1.93	28.5
	7	28.1	2.15	29.6
	14	34.3	2.39	31.0
	28	40.6	2.53	32.5
C50	2	-	-	17.1
	3	33.6	1.75	25.5
	4	-	2.01	28.3
	5	-	2.21	29.1
	7	43.9	2.35	30.9
	14	47.3	2.64	33.7
	28	51.6	2.81	34.5
C60	2	-	-	17.4
	3	52.3	2.03	26.1
	4	-	2.30	28.7
	5	-	2.54	29.3
	7	59.6	2.75	31.7
	14	60.1	3.09	34.4
	28	63.1	3.30	35.2

**Table 6:** Pore characteristic parameters of specimens constrained by steel plates.

Age (Days)		Critical Capillary Diameter (nm)	Porosity (%)	Average Pore Diameter (nm)	Median Pore Diameter (nm)	Pore Distribution (%)			
						<10 nm	10–50 nm	50–100 nm	>100 nm
C30	2	102.54	23.15	26.81	67.51	5.78	26.15	33.16	34.91
	3	91.00	21.11	23.54	63.4	13.21	24.44	36.07	26.28
	4	55.36	20.63	22.81	50.13	21.59	30.16	24.64	23.61
	5	47.27	18.61	21.18	44.94	25.09	41.31	15.69	17.91
	7	35.86	16.68	19.13	44.73	10.24	65.84	11.62	12.29
	14	24.23	15.81	17.63	40.31	13.21	66.24	9.81	10.74
	28	22.71	12.62	14.94	27.91	14.92	70.83	3.72	10.53
C40	2	96.42	94.55	22.91	24.61	64.31	11.44	27.12	29.32
	3	84.65	65.59	21.16	21.55	48.55	14.87	25.64	35.18
	3	51.09	56.65	19.17	21.61	45.54	21.79	25.19	33.81
	5	36.49	45.36	18.73	18.31	42.16	16.63	43.85	25.61
	7	27.95	41.01	18.63	16.74	31.86	12.49	66.01	10.32
	14	23.04	27.37	17.33	16.15	29.31	12.94	66.99	9.11
	28	18.64	21.38	15.35	13.57	20.89	12.97	72.31	4.81
C50	2	93.33	93.33	21.81	23.61	61.87	11.45	26.98	30.48
	3	79.56	65.29	21.02	22.72	56.04	16.39	26.31	34.31
	4	50.38	55.36	18.84	21.61	53.16	14.32	35.19	32.18
	5	36.22	42.7	18.66	19.61	46.15	7.52	53.81	22.16
	7	26.05	32.28	17.52	18.08	38.23	9.73	67.81	11.55
	14	22.03	26.38	15.64	16.31	35.31	10.99	70.31	8.09
	28	17.29	19.62	13.96	13.13	19.39	10.77	74.56	5.51
C60	2	86.67	86.67	21.36	21.81	52.31	12.74	24.16	33.94
	3	71.37	55.15	19.24	20.84	41.43	10.08	69.4	6.37
	4	41.84	41.87	18.61	18.61	33.98	26.86	49.41	11.12
	5	27.42	33.78	17.78	18.03	29.61	24.49	50.16	13.61
	7	21.54	27.84	17.4	15.53	25.15	17.8	68.67	3.07
	14	18.93	23.45	16.87	14.54	24.38	20.33	65.21	4.59
	28	15.15	17.54	13.45	12.67	17.62	28.67	61.74	0.83

**Table 7:** Capillary tension on concrete at four ages (MPa).

	2 Days	3 Days	4 Days	5 Days	7 Days	14 Days	28 Days
C30	1.42	1.60	2.63	3.08	4.06	6.01	6.41
C40	1.51	1.72	2.85	3.99	5.21	6.32	7.81
C50	1.56	1.83	2.89	4.02	5.59	6.61	8.42
C60	1.68	2.04	3.48	5.31	6.76	7.69	9.61

**Table 8:** Elastic modulus comparative analysis.

EM (10^4^ MPa)												
Age (h)	C30			C40			C50			C60		
	$E_{c}$	$E_{t}$	$E_{c}/E_{t}$	$E_{c}$	$E_{t}$	$E_{c}/E_{t}$	$E_{c}$	$E_{t}$	$E_{c}/E_{t}$	$E_{c}$	$E_{t}$	$E_{c}/E_{t}$
48~72	19.15	0.28	68.39	20.3	0.31	65.48	21.3	0.32	66.56	21.75	0.37	58.78
72~96	24.4	4.29	5.69	25.3	5.38	4.70	26.9	5.58	4.82	27.4	5.76	4.76
96~120	26.65	5.63	4.73	27.61	5.7	4.84	28.7	5.95	4.82	29	6.1	4.75
120~168	27.75	7.54	3.68	29.05	7.63	3.81	30	7.85	3.82	30.5	8.06	3.78
168~336	28.7	9.75	2.94	30.3	10.09	3.00	32.3	10.2	3.17	33.05	10.33	3.20
336~672	29.7	10	2.97	31.75	10.64	2.98	34.1	11.31	3.02	34.8	12.0	2.9

**Table 9:** Cracking observation.

	Initial Cracking Time	Average Number of Cracks	Maximum Crack Width	Maximum Crack Length
	(h)		(mm)	(mm)
C30-60	84	3	0.12	33
C30-40	90	5	0.08	31
C30-20	113	5	0.10	35
C40-60	84	10	0.18	41
C40-40	90	8	0.15	33
C40-20	97	9	0.16	35
C50-60	88	16	0.21	43
C50-40	91	9	0.15	36
C50-20	95	12	0.13	45
C60-60	75	5	0.28	63
C60-40	83	14	0.18	45
C60-20	86	15	0.2	35

**Table 10:** Cracking prediction.

	Predicted Cracking Time (h)	True Cracking Time (h)
C30-60	72~96	84
C30-40	72~96	90
C30-20	120~144	113
C40-60	72~96	84
C40-40	72~96	90
C40-20	96~120	97
C50-60	72~96	88
C50-40	72~96	91
C50-20	72~96	95
C60-60	72~96	75
C60-40	72~96	83
C60-20	72~96	86
